# Human Fresh Fibrin Membrane with Bone Morphogenetic Protein-2 (BMP-2) Induces Bone Formation in the Subcutaneous Tissues of Nude Mice

**DOI:** 10.3390/ma14010150

**Published:** 2020-12-31

**Authors:** Keiko Onji, Md. Arafat Kabir, Bowen Zhu, Kenji Yokozeki, Takashi Saito, Toshiyuki Akazawa, Masaru Murata

**Affiliations:** 1 Division of Clinical Cariology and Endodontology, School of Dentistry, Health Sciences University of Hokkaido, 1757 Kanazawa, Tobetsu-cho, Ishikarigun, Hokkaido 061-0293, Japan; onji@takeru-dc.jp (K.O.); t-saito@hoku-iryo-u.ac.jp (T.S.); 2 Division of Oral Regenerative Medicine, School of Dentistry, Health Sciences University of Hokkaido, 1757 Kanazawa, Tobetsu-cho, Ishikarigun, Hokkaido 061-0293, Japan; zhubear@hoku-iryo-u.ac.jp (B.Z.); yokozeki@hoku-iryo-u.ac.jp (K.Y.); murata@hoku-iryo-u.ac.jp (M.M.); 3 Industrial Technology and Environment Research Development, Hokkaido Research Organization, Kita 19-jo Nishi 11-chome, Kita-ku, Sapporo, Hokkaido 060-0819, Japan; akazawa-toshiyuki@hro.or.jp

**Keywords:** blood fibrin, concentrated growth factor (CGF), BMP-2, bone induction

## Abstract

Autologous blood-derived fibrin glue with platelets, called the concentrated growth factor (CGF), can be prepared immediately by only the decided centrifuge without the addition of coagulation factors. Collagen materials combined with recombinant human BMP-2 have been commercially available for clinical use. The fresh CGF is auto-clot with wettability and elasticity, while most collagen membranes are derived from the cow or pig. The fresh CGF has wettability and elasticity, while collagen membranes are dry materials without elasticity. The aim of this study was to observe the microstructures of human CGF membrane and evaluate its behavior as a delivery scaffold of rhBMP-2 in the subcutaneous tissues of nude mice. Twenty-four nude mice (5-week-old, male) were used for the assessment of in vivo ectopic bone formation. Mice were received the CGF membrane as the controls and the CGF/rhBMP-2 membrane as the experimental group in the subcutaneous tissues, and harvested at 7, 10, and 14 days after the graft. Harvested samples were evaluated for the histological examination and the histomorphometric measurement was conducted to compare the residue of the CGF, as well as the new bone. Mature fibrin fibers assembled from multiple fibrillary elements and platelets with the rhBMP-2 membrane induced several bony islands and cartilage without residues of CGF at 14 days, while the CGF membrane alone was almost absorbed at 10 days and failed to induce bone formation at 14 days. These results demonstrated that the fresh, human CGF membrane could contribute to a short-term, sticky fibrin matrix for the delivery of rhBMP-2.

## 1. Introduction

Graft materials from a patient’s own body components have been developed for tissue regeneration. Blood-derived materials and tooth-derived tissues are topics in the bone regeneration field, since they can be used immediately as autologous graft materials [1,2,3,4].

The normal wound healing process starts with blood coagulation, leading to mainly platelet aggregation and the formation of fibrin nets. Blood-derived fibrin with platelets, called a blood clot, plays an important role in both hemostasis and wound healing [5]. Autologous platelet concentrates are generally divided into the following three classifications: Platelet-rich plasma (PRP), platelet-rich fibrin (PRF), and concentrated growth factor (CGF). PRP is a liquid material including concentrated platelets in a small volume of plasma. During the preparation of PRP, a bovine blood-derived activator is needed for coagulation [6,7]. The PRF is prepared solely by centrifugation without the addition of coagulation factors [8,9]. As the second generation, PRF has a solid fibrin consistency, providing a matrix that contains a higher concentration of platelets, leucocytes, and growth factors compared to PRP [2,10,11]. CGF can be prepared within 13 min using only the decided centrifuge that switches the centrifugation speed automatically [12]. In this study, CGF was selected as a blood material from the three platelet concentrates.

Since the discovery of bone morphogenetic proteins (BMPs) [13,14], the delivering carriers of BMPs have been explored. Recombinant human BMP-2 (rhBMP-2), a potent exogenous osteo-inductive cytokine, requires a delivery system that creates optimal conditions for cellular and vascular growth and cellular attachment, as well as a matrix for adsorption [15,16,17,18,19]. Collagenous materials or ceramics combined with rhBMP-2 have been commercially available for clinical use [20,21]. Single component biomaterials such as collagen membranes or ceramics are dry materials without elasticity, while fresh CGF has wettability and elasticity. Although it has been reported that CGF include fibrin and various growth factors, such as platelet-derived growth factors (PDGFs), vascular endothelial growth factors (VEGFs), insulin-like growth factors (IGFs), and transforming growth factor-β1 (TGF-β1) [22,23], the concentration of BMP-2 (10 pg/mL) was significantly lower than that of PDGF (650 pg/mL), TGF-β1, and VEGF. To date, the biological behavior of human CGF membrane has not been clear in vivo, and a graft of human CGF membrane with rhBMP-2 has not been attempted in ectopic sites.

The aim of this study was to observe the microstructures of the human fresh blood-derived CGF membrane and to evaluate the behavior of the human CGF membrane as a delivery scaffold of rhBMP-2 in the subcutaneous tissue of nude mice.

## 2. Materials and Methods 

### 2.1. Ethics Statement

The study design and the consent forms for the procedures performed with the study subjects were approved (no. 150) by the ethical committee for human subjects at the Health Sciences University of Hokkaido with the principles of the Declaration of Helsinki. Blood samples were taken from donor volunteers after obtaining verbal consent. All animal bioassays in this study were conducted in accordance with the Institutional Animal Care and Use Committee of Health Sciences University of Hokkaido.

### 2.2. Preparation of Concentrated Growth Factor (CGF) Membrane 

**Figure 1:**
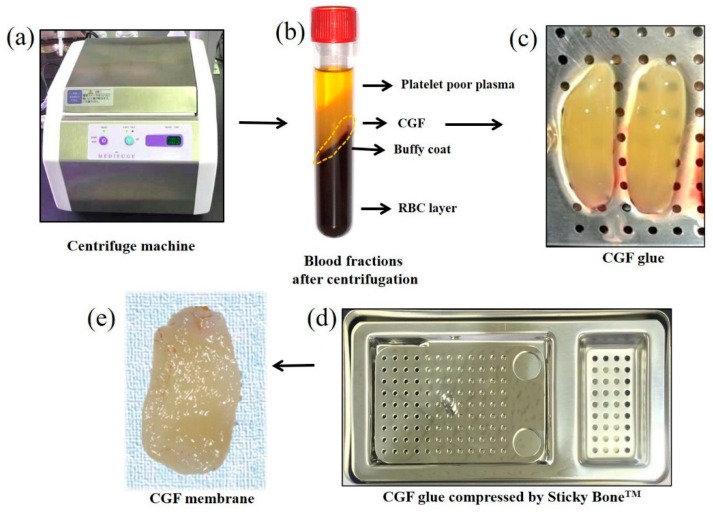
Preparation of concentrated growth factor (CGF) membrane. (**a**) Centrifuge machine (Medifuge^®^, Silfradent Srl, Forli, Italy) used for the preparation of CGF membrane, (**b**) blood fractions after centrifugation: The upper layer represented the clear straw-colored fluid named platelet poor plasma (PPP), the middle layer was a yellow fibrin clot (CGF glue), and the bottom layer represented the red blood cells (RBCs). The buffy coat attached to the yellow glue represented the interface between the lower end of the middle layer and the upper end of the RBC layer. (**c**) A yellow fibrin CGF glue, (**d**) stainless-steel compression device (Sticky Bone^TM^, Jeonju, Korea) to transform the CGF glue into the membrane, (**e**) gross-view of CGF membrane as a rough surface with yellowish-white in color.

Blood samples were collected from three non-smoking, healthy, male volunteers with ages ranging from 25 to 35 years. These donors had no hindrance in daily life nor did they have any systemic diseases. Sixty milliliters of peripheral venous blood was collected in six sterile Vacuette glass tubes without anticoagulant additives using vacuum blood collection tubes, equipped with 18 G needles. The tubes were then immediately centrifuged in a special centrifuge device (Medifuge^®^, Silfradent Srl, Forli, Italy) with the following programs: 30 s acceleration, 2 min at 2700 rpm, 4 min at 2400 rpm, 4 min at 2700 rpm, 3 min at 3000 rpm, and 36 s deceleration and stop (Figure 1). After centrifugation, the upper layer (platelet-poor plasma, PPP) was removed with a sterile syringe, and the middle layer (yellow glue) was collected with sterile tweezers and placed in a sterile petri dish. The lower fraction (red part) of the middle layer was cut as a buffy coat. Half of the yellow CGF glue was pressed by a stainless-steel compression device (Sticky BoneTM, Korea) to prepare the CGF membrane (5 × 5 × 2 mm^3^) (Figure 1). 

### 2.3. Scanning Electron Microscopy (SEM) Analysis 

SEM (JSM-6610LA^®^, Jeol, Tokyo, Japan) was used to investigate the microstructures of the following two fractions: the CGF glue and the CGF membrane. The samples were fixed with 2% neutralized glutaraldehyde for 1 h, and dehydrated serially in 30, 50, 70, 90, and 100% ethanol solutions. The SEM procedures were completed by the critical drying point of the material. Finally, the samples were observed, and the representative images were captured by SEM with an accelerating voltage of 15 kV.

### 2.4. Histological Observation

The CGF membrane was fixed in a 10% neutral phosphate-buffered formalin solution for 24 h, dehydrated with ascending grades of ethanol (50–100%), and processed for paraffin embedding. Later, histological sections with a thickness of 5 µm were prepared and stained with hematoxylin and eosin (H&E).

### 2.5. Animal Experiments

#### 2.5.1. Preparation of Concentrated Growth Factor with Recombinant Human Bone morphogenetic Protein 2 (CGF/rhBMP-2) Membranes

Forty microliters of the rhBMP-2 solution (0.025 g/L) was added to the CGF membrane (5 × 5 × 2 mm^3^). RhBMP-2 was supplied by Astellas Pharma Inc. in Japan. The dose of rhBMP-2 was based on a dose-dependent study in subcutaneous tissues, and 1.0 μg of rhBMP-2 per carrier (5 × 5 × 5 mm^3^) was considered a relatively critical dose for bone induction in subcutaneous tissues [24]. The mixture was kept at 4 °C for 1 h until the in vivo study. As a control group, 40 µL of distilled water was added to the CGF membrane (5 × 5 × 2 mm^3^).

#### 2.5.2. Heterotopic Bioassay

Twenty-four nude mice (5-week-old, male) were used for the assessment of in vivo ectopic bone formation. Ketamine hydrochloride (0.1 mg/kg body weight, Ketalar^®^ 50 mg/mL; Daiichi Sankyo Propharma Co., Ltd., Tokyo, Japan) and xylazine hydrochloride (0.01 mg/kg body weight, Skill pen^®^ 20mg/mL; Interbet Co., Ltd., Tokyo, Japan), diluted with 0.9% NaCl, were used as anesthetics for general anesthesia. Each mouse had two vertical incisions on either side of the back skin. A mouse received one CGF membrane and one CGF/rhBMP-2 membrane. The management of post-operative pain included subcutaneous administration of buprenorphine. The health and behavior of the animals was monitored twice a week in the first week and once a week thereafter. Mice were euthanized with an overdose of anesthesia and cervical dislocation, and the specimens were harvested at 7, 10, and 14 days after the graft.

#### 2.5.3. Tissue Preparation

The specimens were fixed in a 10% neutral phosphate-buffered formalin solution for 24 h, dehydrated with ascending grades of ethanol (50–100%), and processed for paraffin embedding. Later, longitudinal histological sections with a thickness of 5 µm were prepared and stained with hematoxylin and eosin (H&E). Various sectional images were selected through the center of the grafts, and micro-photographs projecting the overall specimens were obtained.

#### 2.5.4. Immunohistochemical Staining for Osteopontin

Following paraffin removal by xylene and rehydration with ethanol, the sections of the CGF/rhBMP-2 membrane at 14 days were treated with 0.3% H_2_O_2_ in 0.01 M phosphate-buffered saline (PBS, pH 7.4) for 20 min at room temperature to inactivate the endogenous peroxidase. They were pretreated with 1% bovine serum albumin (Seikagaku, Tokyo, Japan) in PBS for 20 min at room temperature and incubated in rabbit polyclonal antibody against rat osteopontin (kindly provided by Dr. H. Nakamura) for 12 h at 4 °C [25]. Sections were reacted with the Histofine Simple Stain rat MAX-PO (MULTI; NICHIREI Co., Tokyo, Japan) for 30 min at room temperature. After PBS wash, the immune complexes were visualized using diaminobenzidine (Envision kit; DAKO, CA, USA). Immunostained sections were then counter-stained with hematoxylin, and observed by light microscopy. The non-immune rabbit serum was used as a negative control, rather than the primary antibody. Control sections did not show any specific immunoreactivity.

#### 2.5.5. Elastica Van Gieson Staining 

Following paraffin removal by xylene and rehydration with ethanol, the sections of the CGF/rhBMP-2 membrane at 14 days were pretreated with 1.0% HCl in 70.0% ethanol, and stained with the resorcinol-fuchsin solution for 60 min. The sections were treated with haematoxylin for 3 min, washed in running water, and incubated for 30 min in a picrofuchsia acid solution (1.0% acid fuchsin in aqueous saturated picric acid). Finally, the dehydrated sections were observed by light microscopy.

#### 2.5.6. Histomorphometric Measurement

Explanted tissues were divided into the following three parts: Residue of CGF (acellular matrix), connective tissue (CT), and new bone (NB) with cartilage. Five longitudinal sections were selected from each block, as follows: One section (near the central area), two sections (10 µm from the central), and two sections (40 µm from the central). The areas of CGF residue were outlined by the investigator using the Image J software (Image J 1.46r, developed by the National Institute of Health (NIH), Bethesda, MD, USA). At the end of the measurement, the parameters were calculated as the mean percentage area. A statistical analysis was conducted to compare the areas of CGF, CT, and NB at 7, 10, and 14 days after the graft. All the measurements were performed by one investigator.

#### 2.5.7. Statistical Analysis

All numerical data are presented as the mean ± standard deviation (SD). The statistical significance of the change in response was assessed by a paired *t*-test. Differences were considered significant at *p* < 0.05. The statistical analysis was performed using a Windows computer with the SPSS software version 19 (IBM, Armonk, New York, NY, USA).

## 3. Results

### 3.1. SEM Observation and Gross Histology of Concentrated Growth Factor (CGF) Membrane

**Figure 2:**
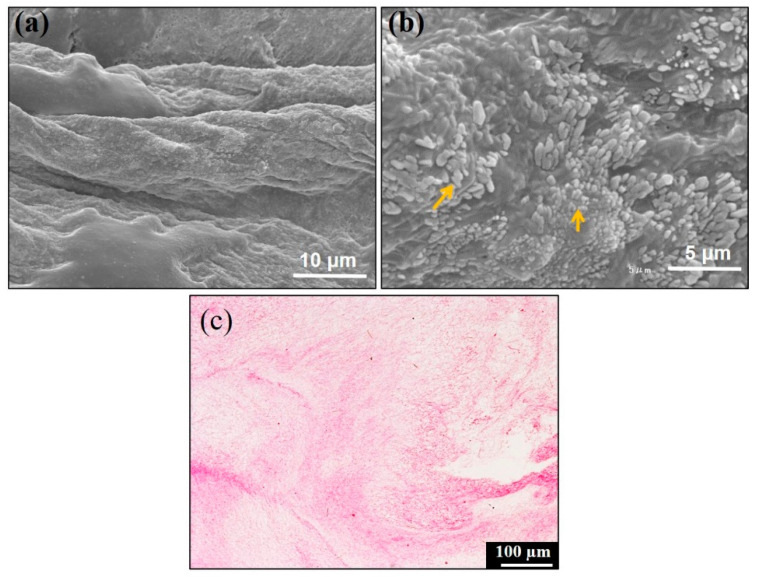
SEM and H&E gross histology of concentrated growth factor (CGF) membrane. (**a**) The CGF layer exhibited mature fibrin fibers assembled from multiple fibrillary elements, (**b**) numerous oblong-shaped platelets (arrows) on the fibrin strand, (**c**) the CGF membrane consisted of fibrin matrices with a non-uniformed structure.

The CGF membrane exhibited mature fibrin fibers assembled from multiple fibrillary elements, and multiple platelets were found on the fibrin strand (Figure 2a,b). The CGF membrane showed an irregular pinkish shape and non-uniform structure due to compression (Figure 2c).

### 3.2. Concentrated Growth Factor (CGF) Membrane in Subcutaneous Tissues

**Figure 3:**
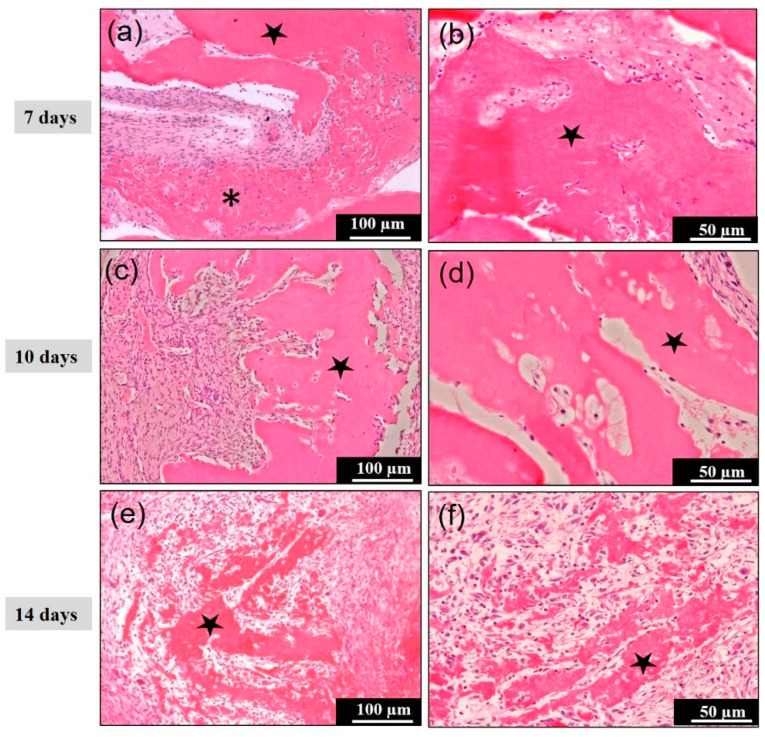
Histological images of concentrated growth factor (CGF) membrane in the subcutaneous tissue. (**a**,**b**) CGF irregular bundles (∗) with fibroblasts and monocytes were mainly observed between various thick and irregular bundles, and an acellular appearance (⋆) was seen inside the thick bundle at 7 days, (**c**) fragmented CGF bundles (⋆) were observed with fibrous connective tissues, (**d**) cellular invasion in the spaces between the bundles at 10 days, (**e**,**f**) the absorbed membrane represented small fragmented residues (⋆) without encapsulation at 14 days.

The membrane revealed a large and small bundle structure at 7 days (Figure 3a). Fibroblasts and monocytes were mainly observed between various thick and irregular bundles, and an acellular appearance was seen inside the thick bundle body at 7 days (Figure 3b). At 10 days, the fragmented bundles were observed with fibrous connective tissues (Figure 3c,d). The mass revealed an irregular surface appearance, and cellular invasion in the spaces between the bundles (Figure 3d). The absorbed membrane represented small fragmented bulks without encapsulation (Figure 3e), and giant cells did not appear at 14 days (Figure 3e,f). These findings showed that the CGF membrane alone failed to induce bone formation. The total volume of the CGF membrane decreased significantly with time (Table 1).

### 3.3. Concentrated Growth Factor with Recombinant Human Bone Morphogenetic Protein 2 (CGF/rhBMP-2) Membrane in Subcutaneous Tissues

**Figure 4:**
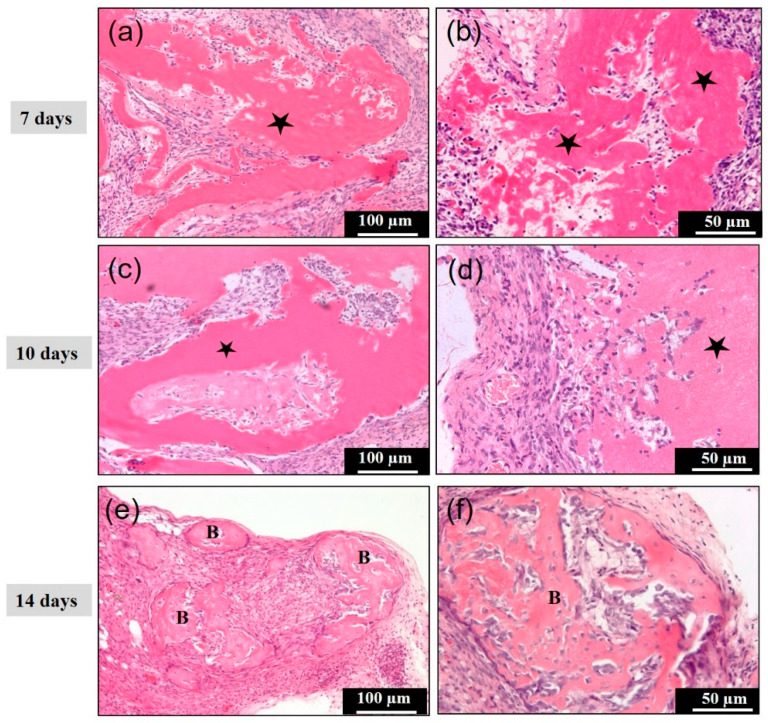
Histological images of concentrated growth factor with the recombinant human bone morphogenetic protein 2 (CGF/rhBMP-2) membrane in subcutaneous tissues. (**a**,**b**) The CGF/rhBMP-2 membrane showed dispersed and disorganized bundles (⋆), and the various bundle surfaces revealed a wavy irregular structure at 7 days, (**c**,**d**) undifferentiated mesenchymal cells and monocytes were observed between the strongly eosin-stained bundles and weakly stained bundles, the bundle area (⋆) represented acellular, and the non-bundle area were cellular at 10 days, (**e**) at 14 days, several induced bone masses (B) were found with fibrous connective tissues and at high magnification, (**f**) the bony island showed a woven bone structure, but not lamellar.

**Figure 5:**
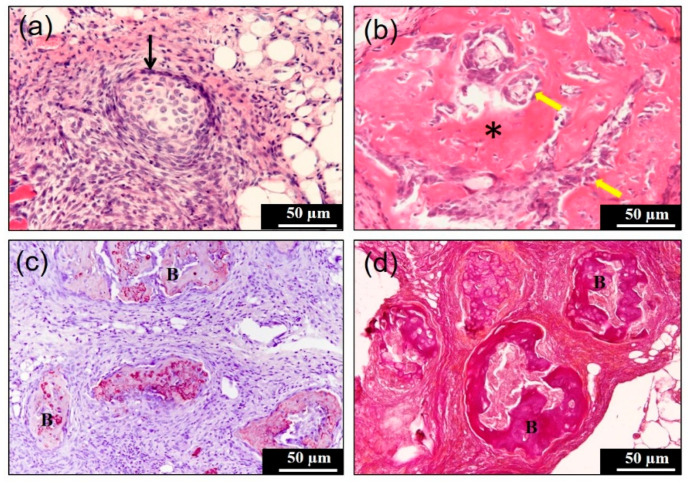
Histological, immunohistochemical, and Elastica van Gieson staining images of concentrated growth factor with the recombinant human bone morphogenetic protein 2 (CGF/rhBMP-2) membrane in subcutaneous tissues at 14 days. (**a**) Cartilage nodule induction (↑) was observed in H&E apart from the induced bone, (**b**) cuboidal osteoblasts (yellow arrows) and the woven bone matrix (∗) with entrapped osteocytes, (**c**) immunoreactivity for osteopontin was detected in the matrix of the induced bone (B), (**d**) the strongly red stained mass (B) represented new bone matrices in Elastica van Gieson staining.

The CGF/rhBMP-2 membrane showed dispersed and disorganized bundles, and the various bundle surfaces revealed a wavy irregular structure at 7 days (Figure 4a,b). An acellular appearance was seen inside the thick bundle (Figure 4b). Undifferentiated mesenchymal cells and monocytes were observed between the strongly eosin-stained bundles and weakly stained bundles at 10 days (Figure 4c), and the bundle area represented acellular, and the non-bundle area were cellular (Figure 4d). At 14 days, several induced bone masses were found with fibrous connective tissues (Figure 4e). At the higher magnification, the bony island showed a woven bone structure, but not lamellar (Figure 4f) meaning an immature bone. On a certain tissue section, the cartilage nodule was induced, apart from the induced bone (Figure 5a), and the cuboidal osteoblasts were lined with capillaries (Figure 5b). The membrane was totally replaced by the bone, cartilage, and fibrous connective tissue at 14 days. The intense immunoreactivity for osteopontin was observed in the induced bone matrix. These positive areas showed a globular appearance and contained large aggregates (Figure 5c,d).

### 3.4. Histomorphometric Assessment 

The average percentage of CGF decreased with time in both groups. The percentage of the CGF membrane group decreased significantly from 7 to 14 days (41.1 ± 0.6% to 10.9 ± 2.4%) (*p* < 0.05). The CGF membrane group without rhBMP-2 did not induce hard tissues during this study. In the CGF/rhBMP-2 group, the percentage of CGF remnants was 13.9 ± 5.2% at 7 days, and CGF remnants were not found at 14 days (Table 1).

## 4. Discussion

The human, fresh CGF membrane with rhBMP-2 induced the bone and cartilage independently at 14 days. The induced bone revealed several islands, and the fresh CGF membrane was replaced by mainly bone and fibrous connective tissues until 14 days. This study did not analyze the release profile of rhBMP-2 from the CGF membrane, but did confirm bony islands induction as positive outcomes for the rhBMP-2 activity.

CGF is composed of cross-linked fibrin strands and a high concentration of platelets that includes various growth factors (PDGF, VEGF, IGFs, and TGF-β1) [22,26]. On the other hand, commercially available ceramics or collagenous materials are 100% inorganic or organic components, without any growth factors. The ceramics are produced by chemical synthesis or produced from the sintered bovine bone, and collagenous materials are derived from bovine or pig skin. The involvement of growth factors released from a patient-own CGF is a completely different point from other biomaterials. Another advantage of CGF in the process of fabrication is its free risk of cross-contamination as the 100% patient-own blood, though the PRP needs bovine thrombin and calcium chloride during the preparation. Unlike the PRF with constant centrifugation, CGF utilizes alternating centrifugation speeds (step by step) from 2400–2700 rpm to isolate a much larger, denser, and rich growth factor fibrin matrix [26]. Moreover, compared to PRF, previous studies showed a better regenerative capacity of CGF with no significant differences in mechanical and degradation properties [26,27]. Since it was reported that bFGF, BMP-2, and TGF-β1 were detected in rabbit-derived CGF and leukocyte- and platelet-rich fibrin (L-PRF) that could promote the proliferation and osteogenic differentiation of rabbit periodontal ligament fibroblasts in vitro [28], bone induction by the CGF alone graft was expected in the in vivo ectopic site. However, bone and cartilage induction did not occur alone in the CGF membrane, in the current study. We believe, therefore, that the growth factors in CGF could stimulate the proliferation of mesenchymal cells, but they do not have the ability to differentiate undifferentiated mesenchymal cells into osteoblasts or chondroblasts. The histological findings were consistent with the report that the concentration of BMP-2 in CGF was fairly low and almost undetectable by ELISA [29]. The addition of 1.0 µg of rhBMP-2 to the CGF membrane (5 × 5 × 2 mm^3^) might provide synergistic effects with several growth factors in CGF for bone and cartilage induction. Recently, the synergistic activity of TGF-β1/BMP-2 or VEGF/TGF-β1/BMP-2 was reported in vitro for osteoblast differentiation [30,31]. Moreover, the combination of BMP-2, VEGF, and TGF-β1 applied according to the time-effect relationship could promote osteogenic differentiation of MC3T3-E1 cells [31]. The reports may support the synergistic effects of rhBMP-2 and CGF-released growth factors for bone induction in this study. In addition, direct bone induction such as an intramembranous ossification was confirmed independent of cartilage induction at 14 days in the CGF/rhBMP-2. The independent inductive findings of bone and cartilage were consistent with the report that the BMP-induced bone and cartilage differentiation depended on the cellular environment derived from the carrier matrix [32,33]. Regarding the choice of animal model, nude mice are ideal for assessing ectopic bone formation, as these immunocompromised animals lack a thymus and do not reject human graft materials [31]. The present study selected subcutaneous tissues of back skin for bioassay, since mice did not have the volume of receiving the human CGF membrane (5 × 5 × 2 mm^3^) in muscles.

BMP-2 is a strong osteoinductive cytokine belonging to the TGF-β superfamily [16]. RhBMP-2 requires biomaterials, which function as sustained-release carriers at the graft site. The present bioassay of human CGF provided the rapid absorption of the CGF from 10 to 14 days and the evidence of bone and cartilage induction at 14 days in the CGF/rhBMP-2 (Table 1). Interestingly, multinucleated giant cells did not appear during the absorbable process of the CGF membrane alone (Figure 3). In our study, we did not observe any trace of the CGF membrane after 2 weeks. This observation is supported by several clinical studies, where clinicians have frequently claimed based on their clinical experiences that fibrin clots were completely degraded within 2 weeks [34,35]. 

Fibrin is well-known to be specifically degraded by plasmin in vivo. Additionally, degradation by other proteases was confirmed in the in vitro study [27]. Therefore, we believe that the CGF membrane was predominantly digested by plasmin and divided into several fragments, but the denser fibrin fibers in the CGF membrane might contribute to the protection of the rhBMP-2 activity from the enzymatic degradation. The inclusion of bioactive factors (VEGF, TGF-, PDGF, BMP-2) into fibrin glue has been studied in tissue engineering, where fibrin serves as the delivery scaffold [36]. Release kinetics showed much greater retention in the fibrin gel after subjection to washes with non-glycosylated BMP-2 versus glycosylated BMP-2 [36]. For clinical use, unique studies related to a controlled release and a dose of rhBMP-2 were published [37,38]. The BMP-2 bound to fibrin glue via a transglutaminase domain with a plasmin sensitive link showed greater retention in the gel compared with the unbound BMP-2 [37]. The heterodimer transglutaminase-BMP-2/7 healed a critical size defect in the rat calvarial bone more efficiently than the homodimer transglutaminase-BMP-2 at the same concentration [38]. The engineering technique will be used to reduce the amount of BMP-2 needed for clinical effect in the near future.

Many studies have considered animal-derived and cross-linked collagen as a qualified candidate for rhBMP-2 delivery in both experimental and clinical researches [39,40]. Our study presents an advanced report related to the combination of rhBMP-2 and human fibrin product (CGF) for ectopic bone induction in the subcutaneous tissue of nude mice. No papers related to the human CGF and blood-related diseases were found in the PubMed search system. From a clinically important point of view, we suggest that patients with blood-related diseases, especially leukemia and malignant lymphoma, or malignant tumors must be excluded from the CGF therapy, since a patient-own CGF consists of white blood cells, platelets, growth factors, and fibrin.

## 5. Conclusions 

The CGF is composed of 100% autologous blood products, and can be prepared immediately for surgery. The CGF membrane alone was almost absorbed under the back skin until 14 days by plasmin degradation. In addition, the CGF/rhBMP-2 membrane induced several bony islands, independent of the cartilage nodule at 14 days. The CGF membrane could support the capability of rhBMP-2 as an absorbable, sticky fibrin matrix. These results demonstrated that the human fresh CGF membrane might act as a short-term biological scaffold for the delivery of rhBMP-2.

## Figures and Tables

**Table 1:** Histomorphometric analysis. The changes in percentage of CGF, connective tissue, and new bone to total volume.

Groups (%)	Explant Period								
	7 Days			10 Days			14 Days		
	CGF	CT	NB	CGF	CT	NB	CGF	CT	NB + Cartilage
CGF Membrane	41.1 ± 0.6	58.8 ± 0.7	0.0	28.1 ± 6.5	70.3 ± 4.3	0.0	10.9 ± 2.4	89.0 ± 2.6	0.0
CGF/rhBMP-2	37.4 ± 2.4	62.5 ± 2.7	0.0 *	22.5 ± 4.3	77.4 ± 4.2	0.0 *	0.0	85.9 ± 4.3	13.9 ± 5.2 *

CGF: Concentrated growth factor; CT: Connective tissues; NB: New bone. The total volume is designated as 100%. Values are mean ± SD; n = 5. * Significant difference *p* < 0.05.

## Data Availability

Data sharing is not applicable to this article.
